# Group D of pulmonary arterial hypertension and its relationship to congenital heart disease: Is there a non-invasive way to predict the unpredictable?

**DOI:** 10.21542/gcsp.2025.5

**Published:** 2025-02-28

**Authors:** Antoine Fakhry AbdelMassih, Alyaa AlAli, Emad Nasr, Eman Hanafy, Musaab Ramsi

**Affiliations:** 1 Pediatric Cardiology, Cairo University, Cairo, Egypt; 2 Pediatric Cardiology, Cardiac Sciences Department, Sheikh Khalifa Medical City, Pure Health Group, Abu Dhabi, UAE; 3 Pediatric critical care, Sheikh Khalifa Medical City, Pure Health Group, Abu Dhabi, UAE; 4 Aswan Heart Centre, Aswan, Egypt; 5 Department Of Mathematics and applied statistics, Faculty of Commerce and Business administration, Helwan University Egypt

## Abstract

Pulmonary arterial hypertension related to congenital heart disease (PAH-CHD), can pose a few challenging therapeutic challenges. PAH related to CHD can be classified into 4 clinical groups: Group A, which includes patients with Eisenmenger syndrome; Group B, which includes patients with severe PAH due to significant shunt lesions with no reversal of the shunt and no cyanosis; Group C, which includes patients with PAH due to small defects whose clinical picture is comparable to that of IPAH (idiopathic PAH) patients; and Group D, which includes patients with persistent PAH following CHD repair. This review aims to shed light on the possible laboratory markers that can predict whether pulmonary arterial hypertension secondary to a congenital heart defect will improve after repair of the defect or will continue to progress because the patient’s PAH is mediated by idiopathic changes and not the shunt lesion itself. This differentiation is crucial for predicting PH prognosis after cardiac repair. See Figure 1 for graphical abstract.

**Figure 1.**
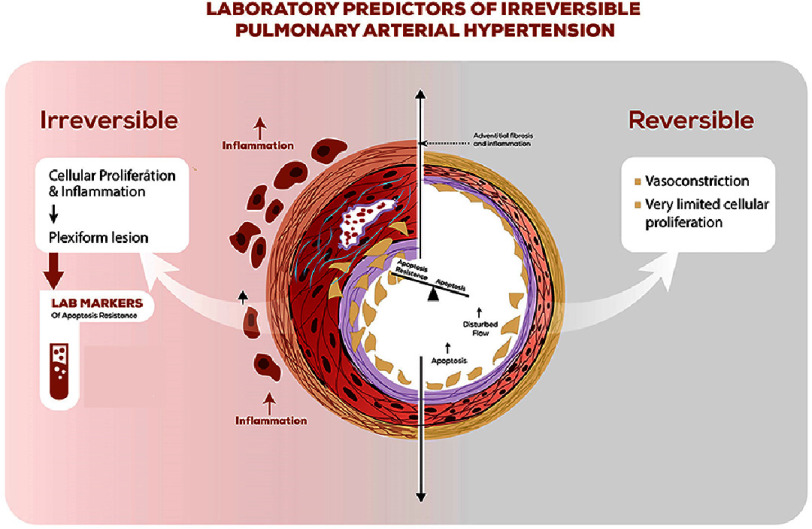
Graphical abstract showing laboratory predictors of irreversible pulmonary arterial hypertension.

## Background

PAH is a common complication of congenital heart disease caused exclusively by shunting lesions. To date, no test has been established that can predict the outcome of pulmonary arterial hypertension and whether PAH will improve after repair of the shunt lesion or will possibly persist. Importantly, no reports to date have successfully identified a distinction parameter that can be used to explore potential factors related to the pathogenesis of PAH in these types of cardiac lesions^1,2^.

The underlying mechanism of PAH-CHD-C and PAH-CHD-D is possibly attributed to the coexistence of idiopathic pulmonary arterial hypertension (IPAH) together with shunt lesions. It is easy to recognize this in Group C; due to the triviality of the associated left-to-right shunt, but it is currently very difficult, if not impossible, to recognize patients categorized into Group D, except retrospectively after the closure of their left-to-right shunt^3^.

The key in prospective identification of PAH-CHD-D is understanding the lung arteriolar landscape in idiopathic PAH and how different it is from early and reversible PAH-CHD. In end-stage (irreversible) PAH, regardless of the underlying etiology, the lung tissue is characterized by concentric laminar hypertrophy and plexiform lesions. While isolated, vasoconstriction or medial hypertrophy is driven by increased pulmonary blood flow and is generally reversible after cessation of the trigger^4^.

The true identification of the reversibility of pulmonary vascular lesions in PAH is either via a vasoreactivity test performed through invasive cardiac catheterization or by invasive lung biopsy. It is illogical to perform any of these procedures routinely before the closure of a large shunt lesion to differentiate between PAH-CHD patients who might respond to CHD repair and those in whom the PAH will progress even after repair^3,6^.

The key factor in the development of malignant plexiform lesions is the loss of apoptotic signals, which leads to excessive and uninterrupted cellular proliferation. Maintaining the balance between apoptosis and apoptosis resistance in endothelial cells has been increasingly established as the turning point between reversible PAH-CHD and irreversible PAH-CHD or, in other words, between early PAH-CHD and other forms of PAH^4^.

Several markers are expressed in endothelial cells and can signal this transition from apoptosis-sensitive to apoptosis-resistant endothelium. P53 is a pro-apoptotic marker that increases PAH-CHD after shunt closure and reflects a good prognosis of PAH regression. However, markers such as Bcl-2 (B-cell lymphoma-2) and survivin are expressed in the lung endothelium of patients with progressive disease, reflecting a shift from apoptosis sensitivity to apoptosis resistance, and others^5,6^.

The aim of this review is to identify potential serum biomarkers that can help in differentiating both states.

## Methodology

To find appropriate primary research articles on this subject, a comprehensive online literature search was conducted using Pubmed, Embase, Clarivate Analytics/Web of Science Core Collection, and Wiley/Cochrane Library, covering publications up to December 2023. The search utilized key terms related to pulmonary hypertension; pulmonary arterial hypertension, IPAH; pulmonary arterial hypertension related to congenital heart disease, biomarkers of reversibility of pulmonary arterial hypertension. The publication date range was set from 2000 to the present. This search yielded a total of 25 publications. Only English-language articles were considered, and the selection included guidelines, clinical trials, reviews, and original research focused on PAH patients. Studies were included if they provided data on biomarkers pertinent to the reversibility of PAH.

The four authors of the study individually examined all potentially relevant titles and abstracts for eligibility. If needed, the full text of articles was reviewed for eligibility. Any disagreements in judgment were resolved through discussions among the four authors. The inclusion criteria for studies were: (1) analysis of potential blood and tissue biomarkers in various forms such as (2) studies involving patients with group 1 pulmonary hypertension (i.e., PAH). Exclusions included: (1) studies not discussing if those biomarkers can discern reversible from irreversible PAH, and (2) certain types of publications like editorials, letters, legal cases, or interviews.

## Results

The literature search identified 15 studies and after exclusion of duplicates, a total of 11 studies were included discussing the role of tissue and serum markers in the detection of reversibility of pulmonary arterial hypertension, especially in sensing apoptosis sensitive vs. apoptosis resistant endothelium.

Six tissue (endothelial) markers (Table 1) have been identified, only one of them has been explored at the tissue and serum levels, namely Urvivin. Caveolin and Cathepsin are linked to uncontrolled smooth muscle proliferation in the pulmonary vascular bed, while the upregulation of the glutathione-transferase MU1 (GSTM1) shields this endothelium from apoptosis, via scavenging oxidative stress.

**Table 1:** Tissue markers of irreversible PAH-CHD.

Authors And reference number in text	Huang et al.^8^	Huang et al.^9^	Li et al.^10^
Positively correlated With pathologic grade	Caveolin-1 Filamin A Cathepsin D	Transgelin	Serum and tissue Urvivin
Negatively correlated with pathologic grade	glutathione-transferase MU1 (GSTM1)		
Tissue sampling	Human subjects	Human subjects	Rats

**Notes.**

Abbreviations PAH-CHD pulmonary arterial hypertension related to congenital heart disease

Serum markers (Table 2) linked with proliferative lesions, and associated with negative outcomes in pulmonary arterial hypertension exerts also their effect via induction of uncontrolled proliferation. Molecules such as PIM-1 and high mobility box group 1, exert their hypertrophy inducing effect by inhibiting cell death and not only by enhancing accelerated mitosis^9–17^.

**Table 2:** Potential serum markers of severity of pulmonary arterial hypertension related to congenital heart disease.

Authors And Reference number in text	Dai et al.^14^	Zhang et al.^13,15^ Huang et al.	Li et al.^16^	Zhu et al.^17^	Li et al.^19^	Gaheen et al.^20^	Daly et al.^21^
Marker	ACE 2 (Angiotensin Converting Enzyme 2)	High mobility group box protein 1	Plasma growth differentiation factor 15 (GDF15)	Pim-1 (provirus integration site for Moloney murine leukemia virus)	Connective tissue growth factor	Copeptin	Endothelin inhibitory hormone
Correlation with severity	Negatively correlated	Positively correlated	Positively correlated	Positively correlated	Positively correlated	Positively correlated	Positively correlated
Other comments				>20.53 ng/mL is indicative of severe pulmonary hypertension			

## Discussion

### Tissue vs. Serum markers, a gap of literature?

The detection of severity of pulmonary arterial hypertension by biomarkers is not new to the literature. Most of the biomarkers validated in literature, such brain natriuretic peptide, are mainly linked to the functional status of patients and the loading effect of PAH on the right ventricle, rather than the reversibility of pulmonary arteriolar lesions.

Tissue markers detected by proteomic analysis of bronchoalveolar lavage or lung biopsy sections, are thought to reflect the grade of pulmonary arteriolar lesions more accurately, but their practicality and the ability to use them in everyday practice is limited. The main aim of exploring tissue markers is to understand the pathogenesis of irreversible PAH, and to determine which of these markers is extensively secreted extracellularly and shed in serum. Unfortunately, the very few studies reporting the biomarkers levels in lung biopsy lesions, did not correlate their tissue and serum levels.

There is no clear distinction between the markers and biomarkers of endothelial cells uncontrolled proliferation, as some are expressed on the cell surface and can also be released into the bloodstream through a process called shedding (such as ACE, VWF, ADAMTS-13, soluble adhesion molecules, and others), and there is no study to date measuring the serum and endothelial levels of potential biomarkers simultaneously particularly in the context of PAH related to CHD^18–20^.

### Suggested study design

We suggest that researchers initiate case-control studies to study the serum levels of the markers across different groups of PAH patients. The proposed study methodology will involve two groups of cases: Group 1: patients with IPAH (whose pathology putatively resembles PAH-CHD-D, and Group 2: patients with PAH-CHD-B. The two groups will have a hemodynamic study (with results of vasoreactivity) and laboratory testing of the markers in Table 2, as well as a lung biopsy for grading of lung pathology.

For sample size calculation, we used the Cochran formula of

\begin{eqnarray*}n= \frac{{n}_{0}}{1+ \left[ \frac{ \left( {n}_{0}-1 \right) }{\mathrm{N}} \right] } \end{eqnarray*}

Where (*N*) refers to the population size which assumed to be 15 to 60 per 1 million with average ∼38 per 1 million, so for 112 million in Egypt, the population assumed to be 4256 observations. Then the sample size is adjusted to 353 patients.

\begin{eqnarray*} \left( {n}_{}= \frac{384.16}{1+ \left[ \frac{ \left( 384.16-1 \right) }{4256} \right] } =352.43\approx 353 \right) \end{eqnarray*}

The biomarkers levels will be expressed using mean standard deviation if normally distributed and using median and interquartile range if skewed. Intergroup differences will be assessed using independent samples T-Test and a *P* value<0.05. Multivariate regression analysis will be implemented to determine the best predictor (among the tested biomarkers) of irreversibility of pulmonary arterial hypertension. A receiver operating characteristic analysis will be conducted to determine the serum level and sensitivity of each marker in differentiating the two study groups^21^.

### Limitation of ethical consideration

The main limitation of this study is ethical considerations. Any biopsy conducted to assess a validated biomarker, regardless of whether it’s part of a clinical trial, is regarded as a clinical biopsy. The main ethical concerns focus on the risks associated with the procedure for the patient and the possible advantages to the patient from analyzing the biospecimen.

### Cost-effectiveness

When selecting a biomarker for research, the decision should be influenced by the scientific objectives and available funding, as costs are a significant consideration. This is particularly relevant for small clinical trials, but in large epidemiological studies involving thousands of participants, expenses can become substantial unless the laboratory methods are streamlined and automated. In fact, larger sample sizes can help reduce the cost per participant, indicating that the biomarker must be accessible and practical to include in the research.

Despite the growing discovery of potential biomarkers in research settings and numerous publications, the use of biomarkers in everyday clinical practice to guide treatment decisions remains quite restricted. While reimbursement choices for new health technologies typically rely on economic evaluations, these assessments for diagnostic and testing technologies, including companion biomarker tests, are reported much less frequently compared to pharmaceuticals. Additionally, there are limited resources in many countries that offer guidance on health economic evaluation methods specifically for co-dependent technologies like companion diagnostics and precision medicine^22,23^.

## Conclusion

PAH-CHD-D patients are an increasingly recognized group of patients in which pulmonary hypertension continues to progress despite the correction of their significant cardiac lesion with pulmonary overflow. Early and noninvasive tests that can predict this subset of patients before correction of the underlying cardiac lesion are still not established in practice and are under investigation in the literature. Using serum markers and angiogenic molecules that indicate apoptosis resistance can potentially change the standard practice within this context. We hope that this review and hypothetical study design will encourage researchers interested in PAH-CHD to start investigating the diagnostic accuracy and cutoff values of these biomarkers.

Constraints include ethical considerations and patients consent regarding lung biopsy, and cost-effectiveness of the study then cost-effectiveness of implementing the wide scale use of expensive biomarkers in clinical practice.

### List of abbreviations

**Table:** 

PAH-CHD	Pulmonary arterial hypertension related to congenital heart disease
IPAH	Idiopathic pulmonary arterial hypertension
BCL	B-cell-lymphoma
GSTM1	glutathione-transferase MU1
Pim-1	provirus integration site for Moloney murine leukemia virus)
GDF15	Plasma growth differentiation factor 15
ACE 2	Angiotensin Converting Enzyme 2

### Authorship and author statements

**Conceptualization:** Antoine AbdelMassih

**Writing-Original draft preparation:** Antoine AbdelMassih, Alyaa AlAli, Emad Nasr, Eman Hanafy, Musaab Ramsi

**Writing-Review & Editing:** Antoine AbdelMassih, Alyaa AlAli, Emad Nasr, Eman Hanafy, Musaab Ramsi

## Acknowledgements

To Sir Magdy Yacoub who read the earliest version of this manuscript and encouraged me to improve it. To patients who inspire me with their resilience and to the medical students and residents in Egypt, UAE, Jordan, India, Iraq, and Palestine, who have been part of my publication journey and enriched it with their valuable input.
